# Formation of hydrogen bonding network of methane sulfonic acid at low degree of hydration (MSA)_m_·(H_2_O)_n_ (m = 1–2 and n = 1–5)

**DOI:** 10.1038/s41598-024-61364-0

**Published:** 2024-05-16

**Authors:** Ahmad Telfah, Z. Charifi, N. latelli, Issam A. Qattan, H. Baaziz, Qais M. Al-Bataineh, A. M. Alsaad, R. F. Sabirianov

**Affiliations:** 1 Nanotechnology Center, The University of Jordan, Amman, 11942 Jordan; 2 Fachhochschule Dortmund University of Applied Sciences and Arts, Dortmund, Germany; 3 Department of Physics, University of Nebraska at Omaha, Omaha, NE 68182 USA; 4 Department of Physics, Faculty of Science, University of M’sila, 28000 M’sila, Algeria; 5 Laboratory of Physics and Chemistry of Materials, University of M’sila, M’sila, Algeria; 6 Department of Chemistry, Faculty of Science, University of M’sila, 28000 M’sila, Algeria; 7 Laboratoire Chimie des Matériaux et des Vivants: Activité, Réactivité, Université Batna1, 05001 Batna, Algerie; 8 Department of Physics, Khalifa University of Science and Technology, P.O. Box 127788, 127788 Abu Dhabi, United Arab Emirates; 9 Leibniz Institut für Analytische Wissenschaften‐ISAS‐e.V., 44139 Dortmund, Germany; 10 Experimental Physics, TU Dortmund University, 44227 Dortmund, Germany; 11 Department of Physical Sciences, Jordan University of Science and Technology, Irbid, 22110 Jordan

**Keywords:** Density functional theory (DFT), Methane sulfonic acid (${\text{MSA}}$), Hydrated clusters, Nuclear magnetic resonance (NMR), IR vibrational bands, Raman vibrational bands, Heat capacity, Physical chemistry, Density functional theory, Chemistry, Materials science, Physics

## Abstract

This study employs ab initio calculations based on density functional theory (DFT) to investigate the structural properties, ^1^H-NMR spectra, and vibrational spectra of methane sulfonic acid (MSA) at low degree of hydration. The findings reveal that energetically stable structures are formed by small clusters consisting of one or two MSA molecules (*m* = 1 and 2) and one or two water molecules in (MSA)_m_·(H_2_O)_n_ (m = 1–2 and n = 1–5).These stable structures arise from the formation of strong cyclic hydrogen bonds between the proton of the hydroxyl (OH) group in MSA and the water molecules. However, clusters containing three or more water molecules (*n* > 2) exhibit proton transfer from MSA to water, resulting in the formation of ion-pairs composed of CH_3_SO_3_^−^ and H_3_O^+^species. The measured ^1^H-NMR spectra demonstrate the presence of hydrogen-bonded interactions between MSA and water, with a single MSA molecule interacting with water molecules. This interaction model accurately represents the hydrogen bonding network, as supported by the agreement between the experimental and calculated NMR chemical shift results.

## Introduction

Water, as a vital solvent for acids and dissociated ions, plays a crucial role in various applications, including fuel cells, condensation reactions, and electrochemical processes^1,2^. Water contains the H-bonding networks that can rearrange according to the changes in the environmental conditions (such as acidity, temperature etc.)^3^. In water solutions, H-bonds are randomly distributed with equal probability for four H-bonds, i.e. two H-donors and two H-acceptors^4^. Methane sulfonic acid (MSA) is a strong organic acid with the formula ${{\text{CH}}}_{3}{{\text{SO}}}_{3}{\text{H}}$ and it is the smallest member of the series of the alkane sulfonic acids. MSA is a main atmospheric oxidation product of ocean-released dimethyl sulfide^5,6^. The MSA consists of ${\text{OH}}$ acidic group, two S=O shares, and one methyl part, is an appropriate model for H-bonding with ${{\text{H}}}_{2}{\text{O}}$ through the sites of ${\text{OH}}$ group and the S=O positions. Investigation of the surface of water structure exposed to adhesions of MSA obviously shows that hydrogen bonding between ${{\text{H}}}_{2}{\text{O}}$ molecules becomes stronger in presence of MSA. Consequently, as the MSA concentration increases, the number of free–OH bonds decreases^7^.

Density Functional Theory (DFT) is a powerful tool for studying and interpreting the dissociation and hydrogen bonding, as well as vibrational and NMR spectra^8–10^. DFT description of intra- and intermolecular hydrogen bonding can be tuned by the choice of the specific functional used to approximate the electronic exchange and correlation (xc) contributions^11^. Bauschlicher et al.^12^ comprehensively analyzed the results obtained from the G2 procedure and DFT methods. He found that the performance of the B3LYP functional is the best among other DFT functional tested. Recently, DiLabio and co-workers examined the effectiveness of the B3LYP method for computing the bond dissociation energies^13^.

Although MSA initial hydration and ionic dissociation have been studied by DFT, IR and Raman spectroscopy of these processes, as well as the behavior of MSA in aqueous solution are not completely resolved. In particular, the dynamics of MSA interaction with water molecules need further investigation. Previous DFT studies were focused on the properties of a single MSA molecule interaction with a few water molecules. Unfortunately, the interaction of MSA molecules in aqueous solution was omitted in these studies.

In NMR no special sample preparation is required, the method is not destructive and permits fast quantitative analysis. NMR spectroscopy fulfills a critical role through its ability to produce unmatched structural information and also to provide data on both intermolecular and intramolecular dynamics^14^. NMR in complement with IR and Raman spectroscopy offers details of MSA interactions in aqueous solutions.

This study investigates dissociation and ionization dynamics of MSA in water based on the structures and properties of the hydration clusters ${\left({\text{MSA}}\right)}_{m}.{\left({{\text{H}}}_{2}{\text{O}}\right)}_{n}$ with *m* ranging from 1 to 2 and *n* ranging from 1 to 5 can be analyzed within density functional theory (DFT). These clusters provide insight into the interactions between MSA and water molecules at the molecular level to analyze the dissociation of MSA due to water interaction. The molecular clusters studied in this work allowing for a deeper understanding of MSA-water clustering characteristics and dynamics of MSA-water clustering for several applications. This approach has been successfully applied to other acid systems as well^3^.

Moreover, in this study, ^1^H-NMR studies in combination with IR spectroscopy. To elucidate the intermolecular dynamics of MSA in aqueous solution we compare our results with DFT based calculations that have been specifically targeting NMR spectra analysis. In this work, we modeled the water clusters ${\left({\text{MSA}}\right)}_{m}\cdot {\left({{\text{H}}}_{2}{\text{O}}\right)}_{n}$ to demonstrate the interactions of MSA with ${{\text{H}}}_{2}{\text{O}}$ molecules in the aqueous phases at the molecular level and then deduce the ^1^H-NMR spectra. We have experimentally conducted ^1^H-NMR spectra corresponding to the molecular ratios calculated using DFT. From the semi-empirical approach, we were able to define the most plausible ${\left({\text{MSA}}\right)}_{m}\cdot {\left({{\text{H}}}_{2}{\text{O}}\right)}_{n}$ clustering by comparing the calculated and the experimental ^1^H-NMR spectra at room temperature.

## Methods

### Experimental

The samples for ^1^H-NMR measurements were prepared by combining a specific number of moles of methane sulfonic acid (MSA) (CH_3_SO_3_H, Mw = 96.11 g/mol) with a specific mole number of distilled water (${{\text{H}}}_{2}{\text{O}}$; DW; Sigma Aldrich) in an argon glovebox. Each prepared sample was transferred to a standard 5 mm NMR tube (Boro-300-5-8; Deutero GmbH). A glass capillary with a diameter of 1 mm and an inner diameter of 0.8 mm was filled with Trimethylsilylpropanoic acid (TSP) in deuterium oxide (${{\text{D}}}_{2}{\text{O}}$; Sigma Aldrich, 99.9% atoms ${\text{D}}$) at a concentration of 0.02 mM and placed inside the 5 mm NMR tube. D_2_O served as the frequency lock, and TSP was used as the chemical shift reference. The NMR samples were degassed by connecting the NMR tubes to a vacuum pump at 800 mb for 50 s while simultaneously sonicating them. The pressure was then raised to normal atmospheric pressure using dry argon. The ^1^H-NMR spectra of the intracellular extracted samples, along with the two reference samples, were acquired using a broadband high-resolution 300.13 MHz NMR Bruker spectrometer Bruker Avance III 300 equipped with a room temperature NMR probe (BBO model-Bruker) at 293 K. The acquisition and processing of NMR spectra were analyzed using the Bruker TopSpin 3.5 software. The ^1^H-NMR spectra were acquired using the standard 90° single-pulse experiment (Bruker pulse sequence ${\text{zg}}$).

### Theoretical approach

All calculations were conducted using the Gaussian 16 Program package^15^. The initial geometries for ${\left({\text{MSA}}\right)}_{m}\cdot {\left({{\text{H}}}_{2}{\text{O}}\right)}_{n} ,\left(m=1-2, n=1-5\right)$ clusters are built up from the optimized structures of MSA and ${{\text{H}}}_{2}{\text{O}}$ using HyperChem7.5 program system. All geometries of the reagents (MSA and ${{\text{H}}}_{2}{\text{O}}$), the clusters ${\left({\text{MSA}}\right)}_{m}\cdot {\left({{\text{H}}}_{2}{\text{O}}\right)}_{n} ,\left(m=1-2, n=1-5\right)$ are structurally and energetically optimized using density functional (DFT) theory with hybrid functional B3LYP^16^. Calculations were performed in 6–311 +  + G (d, p) basis set^17–19^. The optimized structures were confirmed as true minima through vibrational analysis at the same level of theory. The cohesive energies (${E}_{coh}$) were calculated as the difference between the total energy of the cluster and the sum of the total energies of the isolated monomers (MSA and ${{\text{H}}}_{2}{\text{O}}$) confined in the cluster. Zero-point energy is an inherent energy present even in the lowest energy state of a physical system. It arises from the Heisenberg uncertainty principle, which imposes limits on the precision with which the position and momentum of a particle can be known simultaneously. Consequently, even when a particle is in its ground state, it retains a minimum energy known as zero-point energy. In a system composed of multiple atoms exhibiting various normal modes of vibration, the zero-point energy of each mode can be calculated independently. Experimental measurements of zero-point energy at absolute zero temperature are challenging to obtain directly. Therefore, reported experimental values of zero-point energy are typically extrapolated from spectroscopic constants. In this study, zero-point energy-corrected cohesive energies are calculated using the harmonic frequencies obtained from the calculations. Furthermore, the relative free energy, $G$, is calculated at a pressure of 1 bar and temperature of 298.3 K relative to the isolated molecules of MSA and ${{\text{H}}}_{2}{\text{O}}$.

The dissociation of MSA in water can be described by the following equation:

1 $${{\text{CH}}}_{3}{{\text{SO}}}_{3}{\text{H}}+ {{\text{H}}}_{2}{\text{O}}\leftrightarrow {{{\text{CH}}}_{3}{{\text{SO}}}_{3}}^{-}+{{\text{H}}}_{3}{{\text{O}}}^{+}$$

In this equation, ${{{\text{CH}}}_{3}{{\text{SO}}}_{3}}^{-}$ represents the MSA anion and ${{\text{H}}}_{3}{{\text{O}}}^{+}$ is the hydronium ion. ^1^H-NMR spectroscopy allows for the recording of the average chemical shifts (${\updelta }_{av}^{H}$) of the exchangeable proton (^1^H) which can exchange between O–H groups in ${{\text{CH}}}_{3}{{\text{SO}}}_{3}{\text{H}}$ , ${{\text{H}}}_{2}{\text{O}}$ and ${{\text{H}}}_{3}{{\text{O}}}^{+}$. These chemical shifts can be measured experimentally. The calculated ^1^H-NMR chemical shifts of the relevant species can be determined using the following equation. It's important to note that this calculation considers ${{\text{H}}}_{2}{\text{O}}$ and ${{\text{H}}}_{3}{{\text{O}}}^{+}$ are in a very fast exchanging regime and are indistinguishable:

2 $${\updelta }_{av}^{H}= \frac{1}{\left(2{{\text{X}}}_{{{\text{H}}}_{2}{\text{O}}}+{{\text{X}}}_{{{\text{CH}}}_{3}{{\text{SO}}}_{3}\mathrm{H }}\right)}\left(2{{\text{X}}}_{{{\text{H}}}_{2}{\text{O}}}{\updelta }_{{{\text{H}}}_{2}{\text{O}}}+{{\text{X}}}_{{{\text{CH}}}_{3}{{\text{SO}}}_{3}\mathrm{H }}{\updelta }_{{{\text{CH}}}_{3}{{\text{SO}}}_{3}{\text{H}}}\right)$$

In this equation, $2{{\text{X}}}_{{{\text{H}}}_{2}{\text{O}}}$ represents the number of water molecules, and it is multiplied by 2 because it contributes to the chemical shift due to two protons. ${{\text{X}}}_{{{\text{CH}}}_{3}{{\text{SO}}}_{3}{\text{H}}}$ is the number of MSA molecules and contributes to the chemical shift due to a single proton.

The NMR chemical shifts were computed by considering the shielding tensors using both the Continuous Set of Gauge Transformations (CSGT) method^20^ and the Gauge-Independent Atomic Orbital (GIAO) method^21^. In this study, the molecular structures of isolated MSA and ${\left({\text{MSA}}\right)}_{m}\cdot {\left({{\text{H}}}_{2}{\text{O}}\right)}_{n} ,\left(m=1-2, n=1-5\right),$ were fully optimized at the B3LYP/6-311 +  + G(d, p) level of theory. Following optimization, ^1^H-NMR chemical shifts were calculated using the GIAO method^21^ at the same level of theory.

## Results and discussion

**Figure 1:**
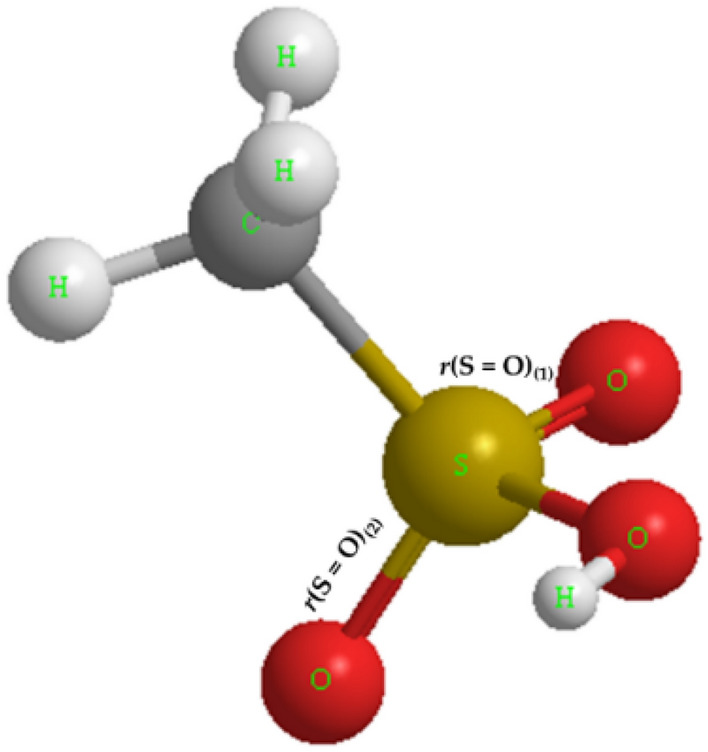
Optimized structure of an isolated MSA molecule using the DFT-B3LYP method.

Figure 1 presents the optimized structure of the isolated MSA molecule, obtained through DFT calculations employing the B3LYP hybrid functional. Geometry optimization involves identifying a stationary point on the potential energy surface, which remains unchanged with temperature variations. Relevant molecular structural parameters are provided in Table 1.

**Table 1:** Comparison of relevant structural parameters of an isolated MSA molecule with corresponding values reported in the literature.

Molecular parameters	This work	Literature B3LYP^7^	Literature MP2^7^
$r\left(C-S\right)$	1.792 Å	1.769 Å	1.770 Å
$r{\left(S=O\right)}_{(1)}$	1.451 Å	1.433 Å	1.444 Å
$r{\left(S=O\right)}_{(2)}$	1.457 Å	1.442 Å	1.450 Å
$r\left(S-O\right)$	1.652 Å	1.606 Å	1.639 Å
$r\left(O-H\right)$	0.969 Å	0.967 Å	0.968 Å
$\angle \left(C-S-O\right)$	97.3°	97.1°	97.7°
$\angle (C-S-O2)$	109.5°	109.5°	109.0°
$\angle (C-S-O1)$	110.7°	110.7°	110.3°

**Figure 2:**
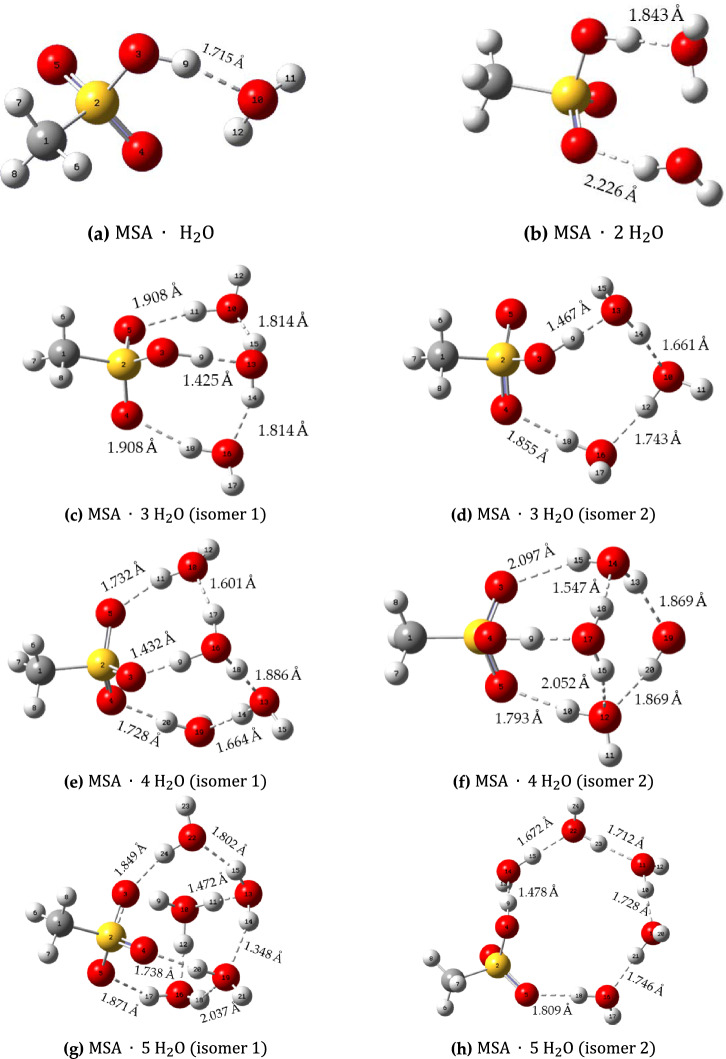
Side views of the equilibrium geometries for ${\left({\text{MSA}}\right)}_{m}\cdot {\left({{\text{H}}}_{2}{\text{O}}\right)}_{n} , (m=1, n=1-5)$, illustrating bond distances.

The variation of bonding as function of water molecule content can be seen in Fig. 2 that shows side views of the equilibrium structures of ${\text{MSA}}.{\left({{\text{H}}}_{2}{\text{O}}\right)}_{n} , (n=1-5)$ together with the bond. The ${{\text{H}}}_{2}{\text{O}}$ molecules form two H-bonds (donor and acceptor). For $n=1 \mathrm{and }2$(Fig. 2a and b), only one lowest free-energy structure is obtained, while for $n=3-5$ (Fig. 2c–h), two isomers exhibit nearly equal free energies.

The monohydrated MSA $\left({\text{MSA}}\cdot {{\text{H}}}_{2}{\text{O}}\right)$ is H-bonded, where the MSA behaves as an H-bond donor and ${{\text{H}}}_{2}{\text{O}}$ behaves as H-bond acceptor. The H-bond distance is 1.715 Å, which is shorter than the water dimers (1.96 Å) (Fig. 2a)^22^. This means that the H-bond between MSA and ${{\text{H}}}_{2}{\text{O}}$ is a very strong H-bond. The O–H covalent bond length of MSA increases from 0.969 to 0.996 Å due to the H-bond interaction between MSA and ${{\text{H}}}_{2}{\text{O}}$. In addition, S–O covalent bond length also decreases from 1.652 to 1.618 Å, while S=O_(1)_ covalent bond length increases to some extent from 1.451 to 1.470 Å. The Gibbs free energy ($G$) has a negative value indicating that the monohydrated MSA is stable as long as dissociation into separate MSA and ${{\text{H}}}_{2}{\text{O}}$ molecules in the gas phase is concerned.

The doubly hydrated MSA $\left({\text{MSA}}\cdot {\left({{\text{H}}}_{2}{\text{O}}\right)}_{2}\right)$ has two H-bond between MSA and ${{\text{H}}}_{2}{\text{O}}$. The first H-bond is the one between MSA donor and ${{\text{H}}}_{2}{\text{O}}$ acceptor with a bond length of 1.843 Å, while the second H-bond is between ${{\text{H}}}_{2}{\text{O}}$ donor and MSA acceptor with a bond length of 2.226 Å (Fig. 2b). The S–O covalent bond length also decreases to 1.600 Å in ${\text{MSA}}\cdot {\left({{\text{H}}}_{2}{\text{O}}\right)}_{2}$. The ${\text{S}}={\text{O}}$ covalent bond lengths are almost unchanged from ${\text{MSA}}\cdot {{\text{H}}}_{2}{\text{O}}$ to ${\text{MSA}}\cdot {\left({{\text{H}}}_{2}{\text{O}}\right)}_{2}$. The cohesive energy increases as two ${{\text{H}}}_{2}{\text{O}}$ molecules are bonded to MSA. This indicates an enhancement in the atom’s arrangement as a crystalline state rather than as a gas state. In addition, increasing the negative value of Gibbs free energy indicates an increase in the structure stability. For ${\text{MSA}}\cdot {\left({{\text{H}}}_{2}{\text{O}}\right)}_{n} , (n=3-5)$, two constitutional isomers (*i.e.*, atoms are bonded together in fundamentally different ways) have nearly equal stability as determined by Gibbs free energy values (difference of around 4.4 kcal/mol). ${\text{MSA}}\cdot {\left({{\text{H}}}_{2}{\text{O}}\right)}_{3}$ has two stable structural isomers. First isomer 1, shown in Fig. 2c, has all three ${{\text{H}}}_{2}{\text{O}}$ molecules bonding directly to MSA, where the 3rd ${{\text{H}}}_{2}{\text{O}}$ molecule is positioned between the first ${{\text{H}}}_{2}{\text{O}}$ and a free O atom of MSA forms two H-bonds. In the second isomer 2, shown in Fig. 2d, ${\text{MSA}}\cdot {\left({{\text{H}}}_{2}{\text{O}}\right)}_{3}$, the third ${{\text{H}}}_{2}{\text{O}}$ molecule is not bonded directly to MSA. Instead, it is bonded to two others ${{\text{H}}}_{2}{\text{O}}$ molecules and located between them, forming a large cyclic hydrogen-bonded network. The two isomers have equal stability, according to the $\Delta G$ values (Table 2). Furthermore, in ${\text{MSA}}\cdot {\left({{\text{H}}}_{2}{\text{O}}\right)}_{4}$ , we find two equally stable isomers as well that can be viewed as modifications of two isomers found in ${\text{MSA}}.{\left({{\text{H}}}_{2}{\text{O}}\right)}_{3}$. First isomer 1, shown in Fig. 2e, can be viewed as the modification of one shown in Fig. 2d where an extra water molecule binds both to MSA and to the cyclic network. The O–H covalent bond length of MSA is considerably augmented to 1.432 Å, and a proton transfer takes place from MSA to ${{\text{H}}}_{2}{\text{O}}$. This results in the development of an ionic pair, ${{\text{CH}}}_{3}{{\text{SO}}}_{3}^{-}\cdots {{\text{H}}}_{3}{{\text{O}}}^{+}$. Second isomer 2 of ${\text{MSA}}\cdot {\left({{\text{H}}}_{2}{\text{O}}\right)}_{4}$, shown in Fig. 2f, can be viewed as modification of one shown in Fig. 2c where an extra water molecule binds to two water molecules with H available for bonding. The O–H covalent bond has a bond length of 1.066 Å, and the H-bond length between MSA and ${{\text{H}}}_{2}{\text{O}}$ is short compared to that of ${\text{MSA}}\cdot {\left({{\text{H}}}_{2}{\text{O}}\right)}_{3}$ of the first isomer. Moreover, the two isomers have the same stability according to the $\Delta G$ values (Table 2). The slight increase in the $\Delta G$ value between ${\text{MSA}}.{\left({{\text{H}}}_{2}{\text{O}}\right)}_{4}$ and ${\text{MSA}}\cdot {\left({{\text{H}}}_{2}{\text{O}}\right)}_{3}$ indicates that any further hydration of ${\text{MSA}}\cdot {\left({{\text{H}}}_{2}{\text{O}}\right)}_{3}$ is thermodynamically reversible. Finally, ${\text{MSA}}\cdot {\left({{\text{H}}}_{2}{\text{O}}\right)}_{5}$ isomer 1 has ion pair of ${{\text{CH}}}_{3}{{\text{SO}}}_{3}^{-}\cdots {{\text{H}}}_{3}{{\text{O}}}^{+}$, which is like ${\text{MSA}}\cdot {\left({{\text{H}}}_{2}{\text{O}}\right)}_{4}$ (Fig. 2g). However, ${\text{MSA}}\cdot {{\text{H}}}_{2}{\text{O}}$ remains H-bonded in ${\text{MSA}}\cdot {\left({{\text{H}}}_{2}{\text{O}}\right)}_{5}$ (isomer 2) (Fig. 2h). Isomer 1 of ${\text{MSA}}\cdot {\left({{\text{H}}}_{2}{\text{O}}\right)}_{5}$ is more stable than isomer 2, as confirmed by the values of $G$ (Table 2). The increase in the negative values of $G$ between ${\text{MSA}}\cdot {\left({{\text{H}}}_{2}{\text{O}}\right)}_{5}$ and ${\text{MSA}}\cdot {\left({{\text{H}}}_{2}{\text{O}}\right)}_{4}$ indicates that the addition of the fifth water molecule is either thermodynamically reversible or to some extent favorable.

**Table 2:** Gibbs free energy ($G, {\text{kcal}}/{\text{mol}}$), cohesive energy (${E}_{coh.}, {\text{kcal}}/{\text{mol}}$), cohesive energy with zero point energy (${E}_{coh.ZPE}, {\text{kcal}}/{\text{mol}}$), molar volume, and molar mass of ${\left({\text{MSA}}\right)}_{{\text{m}}}\cdot {\left({{\text{H}}}_{2}{\text{O}}\right)}_{{\text{n}}} (m=1-2, n=1-5)$.

${\left({\text{MSA}}\right)}_{m}\cdot {\left({H}_{2}O\right)}_{n} [m\cdot n]$	$G [{\text{kcal}}/{\text{mol}}]$	${E}_{tot.} [{\text{kcal}}/{\text{mol}}]$	${E}_{coh.} [{\text{kcal}}/{\text{mol}}]$	${E}_{coh.ZPE} [{\text{kcal}}/{\text{mol}}]$	Molar volume [cm^3^/mol]	Molar mass [g/mol]
${({\text{MSA}})}_{1}$	− 416,907	− 416,927	–	–	63.2	96.0
${({\text{MSA}})}_{2} (1)$	− 833,818	− 833,871	17.4	15.5	114.2	192.0
${({\text{MSA}})}_{2} (2)$	− 833,813	− 833,864	11.1	9.7	107.9	192.0
${({{\text{H}}}_{2}{\text{O}})}_{1}$	− 47,975	− 47,978	–	–	19.5	18.0
${({{\text{H}}}_{2}{\text{O}})}_{2}$	− 95,949	− 95,961	5.8	3.5	31.6	36.0
1 $\cdot$ 1	− 464,883	− 464,917	12.2	9.4	48.9	114.0
1 $\cdot$ 2	− 512,860	− 512,907	25.1	19.9	84.7	132.0
$1\cdot 3 (1)$	− 560,836	− 560,897	37.0	36.0	106.0	150.0
$1\cdot 3 (2)$	− 560,819	− 560,880	19.7	11.9	107.8	150.0
$1\cdot 4 (1)$	− 608,812	− 608,888	50.1	39.8	104.9	168.0
$1\cdot 4 (2)$	− 608,809	− 608,885	47.5	37.0	129.8	168.0
$1\cdot 5 (1)$	− 656,784	− 656,877	61.3	47.0	132.0	186.0
$1\cdot 5 (2)$	− 656,786	− 656,870	55.2	43.4	149.3	186.0
$2\cdot 1$	− 881,789	− 881,855	23.6	18.7	136.4	210.0
$2 \cdot 2 (1)$	− 929,771	− 929,850	40.9	34.4	150.0	228.0
$2\cdot 2 (2)$	− 929,761	− 929,839	29.9	24.1	135.3	228.0
$2 \cdot 3$	− 977,739	− 977,829	42.6	33.8	177.6	246.0
$2 \cdot 4$	− 1,025,711	− 1,025,817	52.5	41.3	165.3	264.0
$2 \cdot 5$	− 1,073,692	− 1,073,807	65.4	52.5	191.4	282.0

Cohesive energy (${E}_{coh.}, {\text{kcal}}/{\text{mol}}$) is calculated ${E}_{coh.}={\left[E{\left({\text{MSA}}\right)}_{m}\cdot {\left({H}_{2}O\right)}_{n}\right]}_{tot}-m*{{\text{E}}\left(\mathrm{ MSA}\right)}_{tot}-n*{{\text{E}}( {H}_{2}O)}_{tot}$. ${E}_{coh.ZPE}$ is calculated in the same relation considering the ZPE energy.

**Figure 3:**
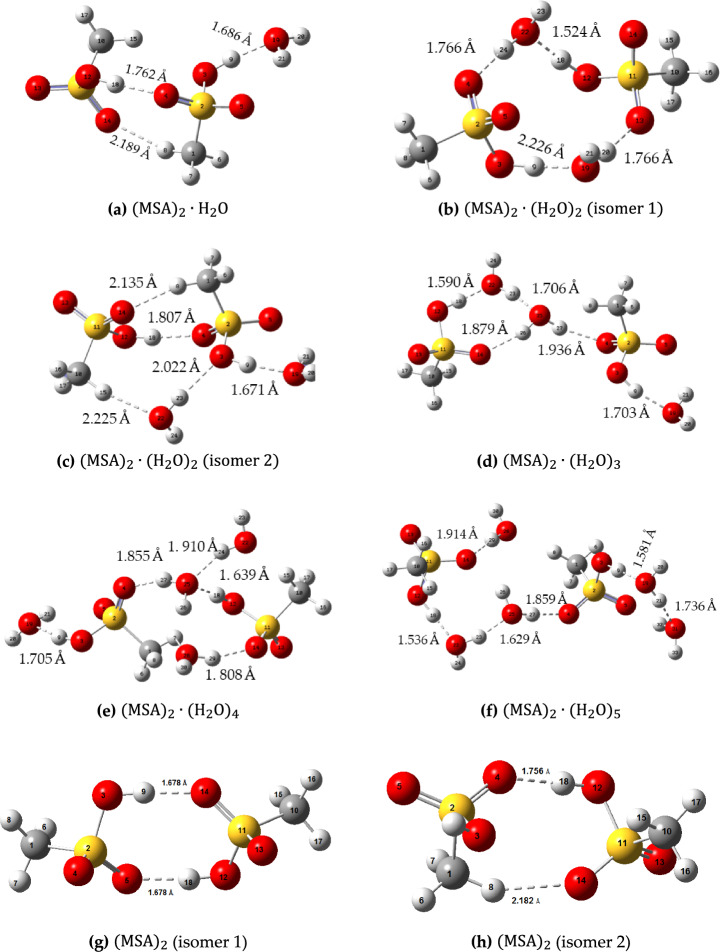
The lateral sights of the equilibrium geometries for ${\left({\text{MSA}}\right)}_{m}\cdot {\left({{\text{H}}}_{2}{\text{O}}\right)}_{n} , (m=2, n=1-5)$. Bond distances are also indicated.

We then turn our attention to consider systems containing two MSA molecules interacting with different ${{\text{H}}}_{2}{\text{O}}$ molecules. Figure 3 shows lateral sights of the equilibrium structures of ${\left({\text{MSA}}\right)}_{2}\cdot {\left({{\text{H}}}_{2}{\text{O}}\right)}_{n} , (n=1-5)$, with bond distances. The ${{\text{H}}}_{2}{\text{O}}$ molecules form two H-bonds (donor and acceptor). For $n=1$ (Fig. 3a), one stable structure is obtained $.{{\text{H}}}_{2}{\text{O}}$ is H-bonded, where the first MSA behaves as an H-bond donor at the OH group with the O-atom of S–O in the second MSA as an H-bond acceptor with a bond distance of 1.762 Å. On the other side, the second MSA behaves as an H-bond donor in the methyl group with the O-atom of S–O in the first MSA as an H-bond acceptor with a bond distance of 2.189 Å. The second MSA behaves as an H-bond donor in the OH group with ${{\text{H}}}_{2}{\text{O}}$ behaves as H-bond acceptor, with an H-bond distance of 1.686 Å.

${\left({\text{MSA}}\right)}_{2}\cdot {\left({{\text{H}}}_{2}{\text{O}}\right)}_{2}$ has two stable isomers (Fig. 3b and c). In the first isomer of ${\left({\text{MSA}}\right)}_{2}.{\left({{\text{H}}}_{2}{\text{O}}\right)}_{2}$ (Fig. 3b), the ${{\text{H}}}_{2}{\text{O}}$ molecule is located between the two MSA molecules, where each ${{\text{H}}}_{2}{\text{O}}$ molecule behaves as an H-bond donor with an H-bond acceptor of MSA from one side and an H-bond acceptor with an H-bond donor of MSA from the other side. In the second isomer of ${\left({\text{MSA}}\right)}_{2}.{\left({{\text{H}}}_{2}{\text{O}}\right)}_{2}$ (Fig. 3c), the two MSA molecules connect to each other via H-bonds. In addition, one ${{\text{H}}}_{2}{\text{O}}$ molecule is positioned between the two MSA molecules, where each ${{\text{H}}}_{2}{\text{O}}$ molecule behaves as an H-bond donor with an H-bond acceptor of MSA from one side and an H-bond acceptor with an H-bond donor of MSA from the other side. Other ${{\text{H}}}_{2}{\text{O}}$ molecule behaves as an H-bond acceptor with the H-bond donor of MSA. Figure 3d–f also show the two MSA molecules which are connected to 3, 4 and 5 molecules of ${{\text{H}}}_{2}{\text{O}}$ which positioned allowing to each ${{\text{H}}}_{2}{\text{O}}$ molecule behaves as an H-bond donor with an H-bond acceptor of MSA/water from one side and an H-bond acceptor with an H-bond donor of MSA/water from the other side. After proton transfer from the first MSA to the second MSA, where MSA behaves as an H-bond donor with an H-bond acceptor of MSA from one side and an H-bond acceptor with an H-bond donor of MSA from the other side, implies that direct acid–acid interactions are possible leading to two different favorable isomers (Fig. 3g and h). The dimeric association of two MSA molecules (Fig. 3g) appears to be more stable due to formation of two strong H-bonds.

**Figure 4:**
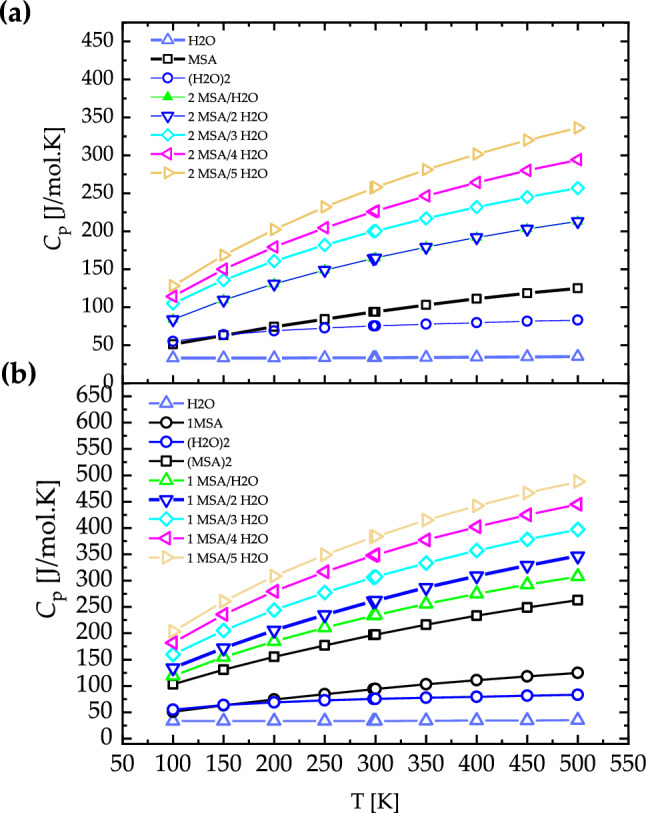
Heat capacity $({C}_{p}, [{\text{J}}/{\text{mol}}.{\text{K}}])$ of isolated MSA and ${\left({\text{MSA}}\right)}_{{\text{m}}}\cdot {\left({{\text{H}}}_{2}{\text{O}}\right)}_{{\text{n}}}$, ($n=1-5$), (**a**) $m=1$ and (**b**)$m=2$ calculated by DFT method.

Figure 4 shows the calculated heat capacity for ${\left({\text{MSA}}\right)}_{{\text{m}}}\cdot {\left({{\text{H}}}_{2}{\text{O}}\right)}_{{\text{n}}} (m=1-2, n=1-5)$ binary systems. The heat capacity of the isolated MSA increases from 50 to 164 [J/mol K] as MSA temperature is increased from 100 to 500 K. At lower temperature, the translation and rotation of the molecule are the major contributions to the heat capacity. At a higher temperature, the vibrations contribute more to the heat capacity. This leads to an increase of the heat capacity as the temperature is increased^23^. For ${\left({\text{MSA}}\right)}_{{\text{m}}}\cdot {\left({{\text{H}}}_{2}{\text{O}}\right)}_{{\text{n}}} ,(m=1-2, n=1-5)$ binary systems, heat capacity increases as the number of MSA and ${{\text{H}}}_{2}{\text{O}}$ molecules are increased. For instance, the heat capacity of ${\text{MSA}}\cdot {\left({{\text{H}}}_{2}{\text{O}}\right)}_{5}$ increases from 128 to 441 [J/mol K] as the temperature is increased from 100 to 500 K, while the heat capacity of ${\left({\text{MSA}}\right)}_{2}\cdot {\left({{\text{H}}}_{2}{\text{O}}\right)}_{5}$ increases from 203 to 623 [J/mol K] as the temperature is increased from 100 to 500 K. The formation of intermolecular hydrogen bonding is the main reason for increasing the heat capacity^23^. In addition, increasing the number of MSA and ${{\text{H}}}_{2}{\text{O}}$ molecules increases the molecular vibration and thus, increases the heat capacity^24^. The heat capacity of MSA-water solution increases as the number of MSA or water molecules increases. As the water content increase resulting in a larger and more complex hydrogen bonding network which are directly proportional to the potential energy stored in the network. At higher temperatures, more molecules have sufficient thermal energy to overcome the potential energy barriers in hydrogen bonding. Thus, the size of the hydrogen bonding network increases with temperature, leading to a larger potential energy stored in the system^25,26^. The increase in potential energy stored in the hydrogen bonding network leads to a larger heat capacity of the system. The potential energy stored in the hydrogen bonding network can be converted into thermal energy as the system is heated. Therefore, an increase in the size of the hydrogen bonding network leads to an increase in the amount of potential energy stored in the system, which can be released as heat, resulting in a larger heat capacity. A molecular dynamics study on the effects of water on the structural and thermodynamic properties of methanesulfonic acid found that an increase in the number of water molecules led to a larger hydrogen bonding network and a higher heat capacity of the system confirming the results obtained in this work linking the increase in the water molecules per sulfonic acid molecule and heat capacity^25^.

**Figure 5:**
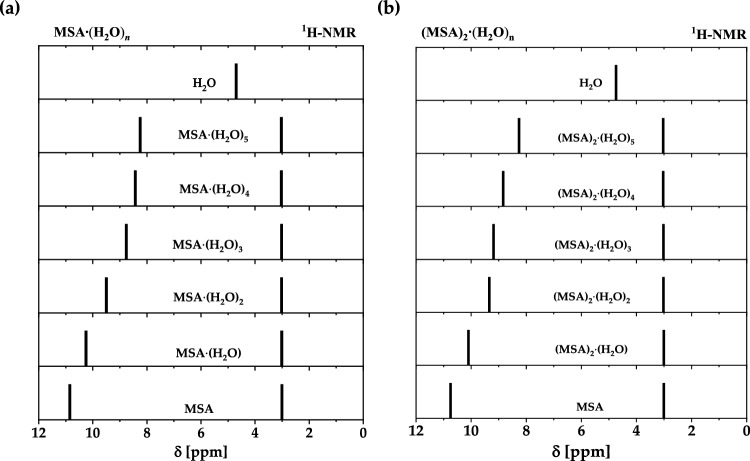
Theoretical ^1^H-NMR spectra based on the calculated chemical shifts of isolated MSA, water and ${\left({\text{MSA}}\right)}_{{\text{m}}}\cdot {\left({{\text{H}}}_{2}{\text{O}}\right)}_{{\text{n}}}$ ($n=1-5$), (**a**) $m=1$ and (**b**) $m=2$.

Figure. 5a and b shows the theoretical ^1^H-NMR spectra of an isolated MSA and ${\text{MSA}}{\cdot \left({{\text{H}}}_{2}{\text{O}}\right)}_{n}$ and ${\left({\text{MSA}}\right)}_{m}\cdot {\left({{\text{H}}}_{2}{\text{O}}\right)}_{n} , (n=1-5)$ based on the calculated chemical shifts. The hydroxyl group (OH) group in the ${\text{MSA}}\cdot {\left({{\text{H}}}_{2}{\text{O}}\right)}_{n}$ systems are the superposition between the OH protons of ${{\text{H}}}_{2}{\text{O}}$, ${{\text{H}}}_{3}{{\text{O}}}^{+}$, and the ${\text{MSA}}$. This leads to an abrupt reduction in the chemical shifts. However, the chemical shift of the methyl protons is slightly affected by adding water molecules to the system due to the change in the chemical of the environment.

Figure 5b shows the theoretical approach of ^1^H-NMR for an isolated MSA and ${\left({\text{MSA}}\right)}_{2}\cdot {\left({{\text{H}}}_{2}{\text{O}}\right)}_{n} , \left(n=1-5\right)$ clusters. The OH group in the ${\left({\text{MSA}}\right)}_{2}\cdot {\left({{\text{H}}}_{2}{\text{O}}\right)}_{n}$ systems are the superposition between the OH protons of ${{\text{H}}}_{2}{\text{O}}$, ${{\text{H}}}_{3}{{\text{O}}}^{+}$, and the ${\text{MSA}}$. This leads to an abrupt decrease in the chemical shifts. However, the chemical shift of the methyl protons is slightly affected by adding water molecules to the system. According to Fig. 3a the dimeric association is broken and ${\text{O}}\cdots {\text{H}}\cdots {\text{C}}$ is formed, causing noticeable chemical shift, typically, hydrogen bonded dimeric association is expected (with two symmetric ${\text{O}}\cdots {\text{H}}\cdots {\text{OH}}-{\text{bonds}}$ as shown in Fig. 3g).

**Figure 6:**
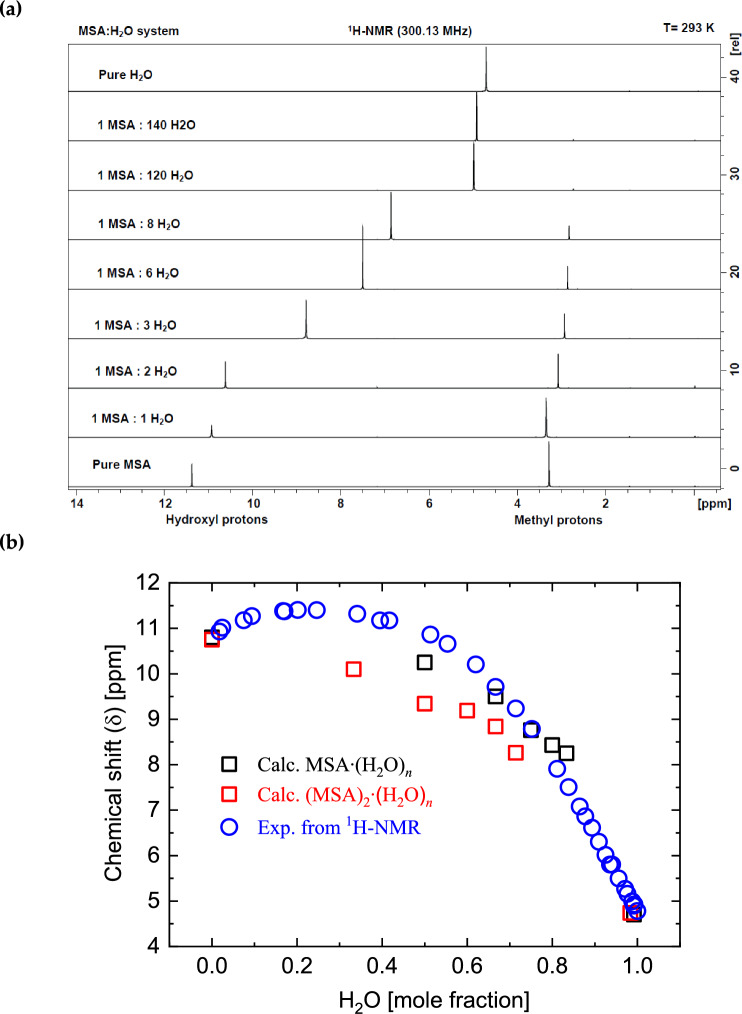
(**a**) Selected ^1^H-NMR spectra of ${\text{MSA}}\cdot {({{\text{H}}}_{2}{\text{O}})}_{n}$, $\left(n=0-140\right)$, and pure water taken at 300.13 MHz and 293 K. (**b**) Chemical shifts deduced from the experimental NMR spectra in (a) and the calculated NMR chemical shifts in ${\text{MSA}}\cdot {({{\text{H}}}_{2}{\text{O}})}_{n}$ and ${({\text{MSA}})}_{2}\cdot {({{\text{H}}}_{2}{\text{O}})}_{n}$ $\left(n=0-5\right)$. Selected spectra are illustrated for clarity, while experimental data cover all points in (b).

Figure 6a displays selected ^1^H-NMR spectra of ${\text{MSA}}\cdot {({{\text{H}}}_{2}{\text{O}})}_{n}$ , $\left(n=0-140\right)$, along with pure water, acquired at 300.13 MHz and 293 K. In Fig. 6b, the chemical shifts deduced from the experimental NMR spectra and the calculated NMR chemical shifts in ${\text{MSA}}\cdot {({{\text{H}}}_{2}{\text{O}})}_{n}$ and ${({\text{MSA}})}_{2}\cdot {({{\text{H}}}_{2}{\text{O}})}_{n}$ $\left(n=0-5\right)$ are presented. Selected spectra are shown for clarity, while all spectra are covered. Figure 6b also presents a comparison between experimentally deduced and calculated chemical shifts of the 1H-NMR as a function of the mole fraction of water.

The chemical shift of the hydroxyl group resonance peak towards the lower chemical shifts indicates the existence of H-bond networking between MSA and ${{\text{H}}}_{2}{\text{O}}$ resulting in ${{\text{H}}}_{3}{{\text{O}}}^{+}$. There is overall agreement between experimental and theoretical values of chemical shift as can be seen from Fig. 6b. We attribute a small discrepancy to the formation of hydronium ion at low water concentration. The chemical shift of hydronium ion is ~ 12–13 ppm^27^, that is larger than one measured in pure MSA. The formation of hydronium ion at low water concentration causes a slight increase in hydroxyl group chemical shift as molar content of water increases. Theoretical calculations do not include hydronium atom formation and therefore do not show the increase in chemical shift at low mole fraction. However, the $\mathrm{MSA }\cdot {\left({{\text{H}}}_{2}{\text{O}}\right)}_{n}$, $\left(n=1-5\right)$ is more fitting to the experimental data. On this side, the higher concentration of MSA contains higher interactions among MSA molecules and therefore can be described better by the model where two molecules of MSA are in rule.

**Figure 7:**
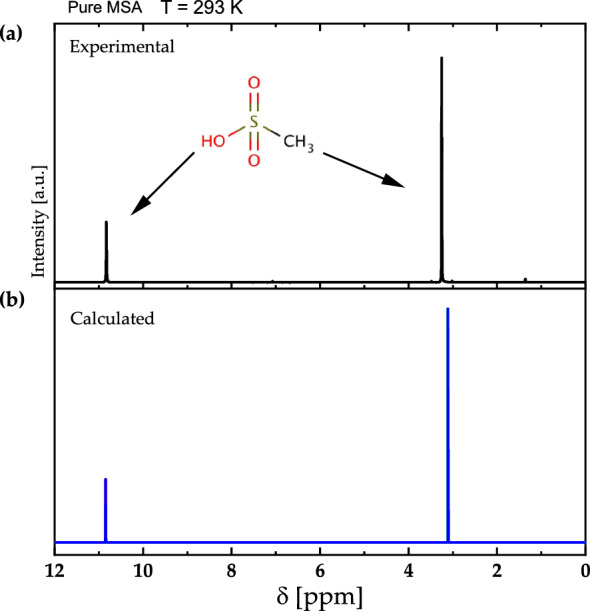
(**a**) The experimental and (**b**) calculated ^1^H-NMR spectra of pure MSA at 600.13 MHz of an isolated MSA molecule at 293 K.

Figure 7a and b display the experimental and calculated ^1^H-NMR spectra of pure MSA, respectively, utilizing the optimized structure acquired in Fig. 3. The comparison of the two resonance peaks with chemical shifts of 3.35 and 10.93 ppm corresponding to the protons in methyl and ${\text{OH}}$ groups, respectively, clearly shows a good agreement between the theoretical and experimental spectrum. The slight difference is attributed to the fact that theoretical approach treats MSA as an isolated species neglecting the interactions between MSA molecules that are present in the experimental measurements. The splitting appeared in the experimental NMR signal of ^1^H in methyl group is the spectral multiplet attributed to the dipolar coupling which has not been considered in calculations. While the splitting appeared in the ^1^H in the hydroxyl group is associated with the sampling rate and digitizing imperfection since no spectral multiplet could appear in the ^1^H in hydroxyl group because the ^1^H is exchangeable between MSA, ${{\text{H}}}_{2}{\text{O}}$, ${\text{OH}}$ and ${{{\text{H}}}_{3}{\text{O}}}^{+}$ in a very fast rate with respect to the NMR detection. The exchange can only be seen as a resultant in amplitude and chemical shift^28^. The calculated NMR spectrum is generated from the ^1^H chemical deduced from the DFT modeling, while the signal magnitude is calculated from the fitting of the peak at the calculated chemical shifts. The fitting to the Lorentzian function (typical function described NMR line in homogeneous liquid) is considering by setting the area under the fitted line to be equal to the ration between the number of ^1^H in the moieties that forms the NMR signal in each NMR spectrum.

**Figure 8:**
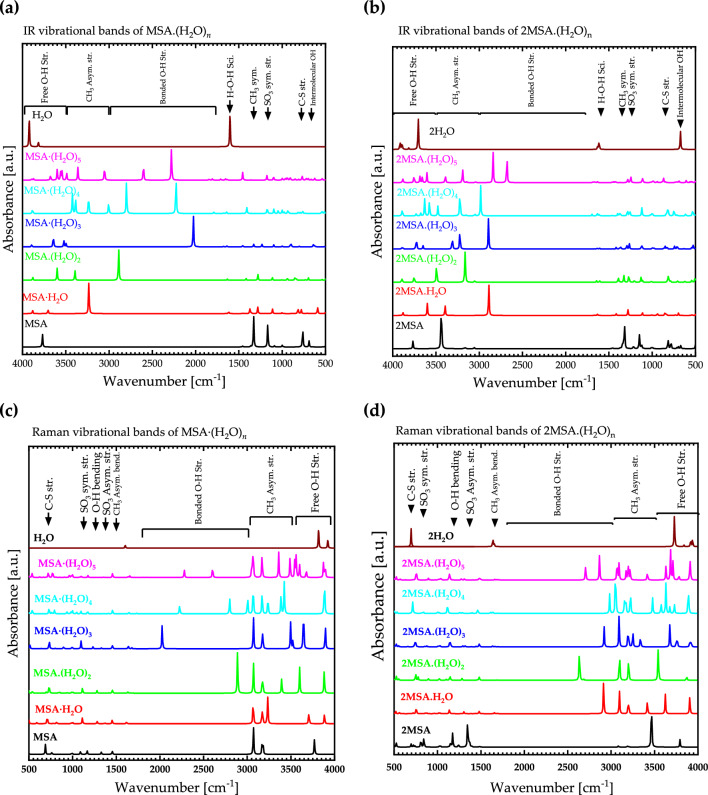
Theoretical IR and Raman spectra for ${{\text{H}}}_{2}{\text{O}}$ and ${\text{MSA}}\cdot {({{\text{H}}}_{2}{\text{O}})}_{n}$ (**a** and **c**) and for $2{{\text{H}}}_{2}{\text{O}}$ and ${\left({\text{MSA}}\right)}_{2}.{\left({{\text{H}}}_{2}{\text{O}}\right)}_{{\text{n}}}$ (**b** and **d**) where $n=1-5$.

The calculated IR vibrational bands of isolated MSA and ${{\text{MSA}}}_{m}\cdot {\left({{\text{H}}}_{2}{\text{O}}\right)}_{{\text{n}}} (n=1-5, m=1-2)$ are presented in Figs. 8a and b for m = 1 and 2, respectively. For isolated MSA, the IR vibrational bands are located at 688 ${{\text{cm}}}^{-1}$, 760 ${{\text{cm}}}^{-1}$, 1168 ${{\text{cm}}}^{-1}$, 1328 ${{\text{cm}}}^{-1}$, and 3768 ${{\text{cm}}}^{-1}$, corresponding to intermolecular ${\text{OH}}$, C–S stretching, ${{\text{SO}}}_{3}$ symmetrical stretching, ${{\text{CH}}}_{3}$ symmetrical stretching, and O–H stretching, respectively. These data are in good agreement with existing literature data^29^. In the gas phase, the symmetric and asymmetric O–H stretches of water are at 3657 ${{\text{cm}}}^{-1}$ and 3756 ${{\text{cm}}}^{-1}$, respectively, and these frequencies redshift to 3404 ${{\text{cm}}}^{-1}$ in the bulk due to hydrogen bonding^30^. In the calculations performed in the gas phase, the frequencies of O–H in pure water in Fig. 8a and b are around 3760 ${{\text{cm}}}^{-1}$, demonstrating agreement with published theoretical data. The vibrational band of O–H in pure MSA appears at around 3600 ${{\text{cm}}}^{-1}$. However, the peak shifts toward higher frequencies with the addition of water molecules to the system due to hydrogen bonding, resulting in a blue shift. The maximum shifts occur in the regime where one MSA molecule is combined with three water molecules.

In the case of two MSA molecules, the shift to higher frequency is also observed, with maximum shifts occurring when there are two to three water molecules per two MSA molecules. Wang^31^ have investigated clustering of Hydrated Methane Sulfonic Acid $({{\text{CH}}}_{3}{{\text{SO}}}_{3}{\text{H}}\cdot ({{{\text{H}}}_{2}{\text{O}})}_{n} , (n = 1-5))$ and observed the formation of strong hydrogen bonds that causes strong red shift of O–H vibrational stretching mode which is in agreement with this study. It also greatly enhances the IR transition of these modes when the ${\text{OH}}$ bond participates in hydrogen bond formation. From a theoretical point of view, intermolecular interactions are typically explored via a supermolecular approach. In such an approach, the interaction enthalpy is accurately estimated between the energies of a supermolecular complex and its fragments^32^.

Figure 8c and d shows theoretical Raman spectra for ${{\text{H}}}_{2}{\text{O}}$ and ${\text{MSA}}\cdot {({{\text{H}}}_{2}{\text{O}})}_{n}$ as well as $2{{\text{H}}}_{2}{\text{O}}$ and ${\left({\text{MSA}}\right)}_{2}.{\left({{\text{H}}}_{2}{\text{O}}\right)}_{{\text{n}}}$ where $n=1-5$. The isolated MSA has Raman vibrational bands located at 688 ${{\text{cm}}}^{-1}$, 1088 ${{\text{cm}}}^{-1}$, 1168 ${{\text{cm}}}^{-1}$, 1328 ${{\text{cm}}}^{-1}$, 1456 ${{\text{cm}}}^{-1}$, 3072 ${{\text{cm}}}^{-1}$, 3184 ${{\text{cm}}}^{-1}$, and 3768 ${{\text{cm}}}^{-1}$ corresponding to C–S stretching, ${{\text{SO}}}_{3}$ symmetrical stretching, O–H bending, ${{\text{SO}}}_{3}$ asymmetrical stretching, ${{\text{CH}}}_{3}$ asymmetrical bending, ${{\text{CH}}}_{3}$ asymmetrical stretching, and O–H stretching, respectively.

${\text{MSA}}.{\left({{\text{H}}}_{2}{\text{O}}\right)}_{{\text{n}}}$ binary systems have new vibrational bands in IR and Raman spectra in (1500–3000) cm^-1^ spectral range, corresponding to O–H stretching bonds which are in good agreement with literature^30^.

**Figure 9:**
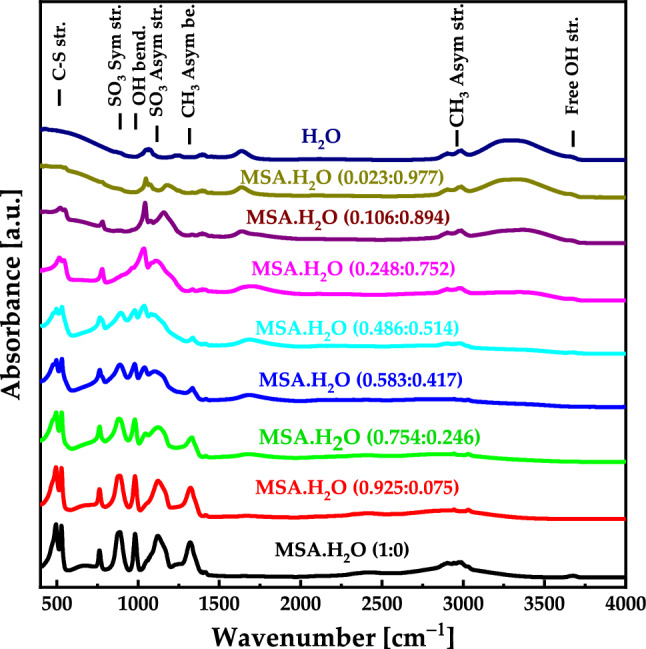
Experimental IR spectra for ${\text{MSA}}\cdot {{\text{H}}}_{2}{\text{O}}$ with different mole fraction.

The experimental IR vibrational bands of pure MSA and ${\text{MSA}}\cdot {{\text{H}}}_{2}{\text{O}}$ with different mole fraction are presented in Figs. 9. For pure MSA, the IR vibrational bands are located at 530 ${{\text{cm}}}^{-1}$, 892 ${{\text{cm}}}^{-1}$, 886 ${{\text{cm}}}^{-1}$, 983 ${{\text{cm}}}^{-1}$, and 1120 ${{\text{cm}}}^{-1}$, 1325 ${{\text{cm}}}^{-1}$, 2930 ${{\text{cm}}}^{-1}$, and 3678 ${{\text{cm}}}^{-1}$, corresponding to C–S stretching, ${{\text{SO}}}_{3}$ symmetrical stretching, O–H bending, ${{\text{SO}}}_{3}$ asymmetrical stretching, ${{\text{CH}}}_{3}$ symmetrical bending, ${{\text{CH}}}_{3}$ symmetrical stretching, and free O–H stretching, respectively.

Comparing the theoretical and experimental IR results, we find good agreement with existing literature data^29^ as well as the calculated data (Fig. 8a and b). The vibrational band of stretching O–H in pure MSA appears at around 3678 ${{\text{cm}}}^{-1}$ and with the addition of water molecules to the system, the peak shifts toward higher frequencies due to hydrogen bonding. It's worth noting that some calculated vibrational and stretching IR frequencies may not appear in the experimental spectra due to quenching, as the simulations in DFT were performed in the gas phase. Additionally, in experimental settings, the molecule is typically in a bulk form, which may result in the suppression of certain vibrational modes (and therefore spectral lines).

**Figure 10:**
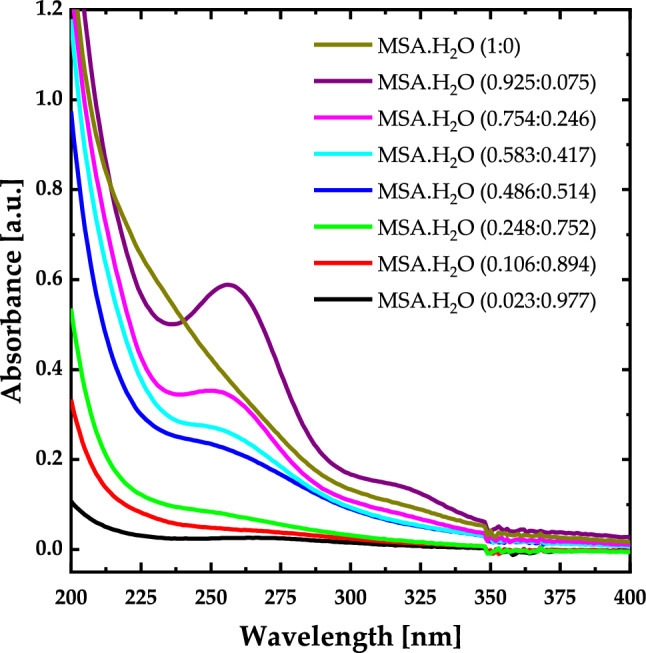
Experimental UV–Vis spectra for ${\text{MSA}}\cdot {{\text{H}}}_{2}{\text{O}}$ with different mole fraction.

The experimental UV–Vis absorbance spectra of pure MSA and ${\text{MSA}}\cdot {{\text{H}}}_{2}{\text{O}}$ with different mole fraction are presented in Figs. 10. The absorbance spectrum for pure MSA exhibits a sudden increasing below 400 nm, representing the $\pi -{\pi }^{*}$ transition bands of MSA. Introducing H_2_O into MSA leads to appear an absorption band at 257 nm, resulting from the hydrogen bonding between MSA and H_2_O.

## Conclusions

The structures and energetics of hydrated clusters of methane sulfonic acid ${\left({\text{MSA}}\right)}_{{\text{m}}}\cdot {\left({{\text{H}}}_{2}{\text{O}}\right)}_{n} , \left(m=1-2, n=1-5\right)$ are investigated using ^1^H-NMR and analyzed in detail using DFT. Consistent with previous findings, strong hydrogen bonding occurs between the ${\text{OH}}$ group of ${\text{MSA}}$ and ${{\text{H}}}_{2}{\text{O}}$ molecules. The ${\left({\text{MSA}}\right)}_{m}\cdot {\left({{\text{H}}}_{2}{\text{O}}\right)}_{n} , \left(m=1-2, n=3-5\right)$ clusters exhibit multiple stable isomers due to the availability of multiple binding sites on MSA and the hydrogen bonding capability of water at low degree of hydration. As the cluster size increases to three or more water molecules, the MSA anion undergoes proton transfer to water, resulting in the formation of ionic ${{\text{CH}}}_{3}{{\text{SO}}}_{3}^{-}$ and ${{\text{H}}}_{3}{{\text{O}}}^{+}$ species within the clusters. The energy difference between the ionic and neutral isomers is not significantly pronounced for ${\text{MSA}}\cdot {\left({{\text{H}}}_{2}{\text{O}}\right)}_{n} , \left(m=1-2, n=3-5\right)$ clusters. Hence, a coexistence of ionic and neutral isomers is predicted by DFT and confirmed by experimental ^1^H-NMR spectra, particularly through the observed chemical shifts of exchangeable protons in MSA, water, ${{\text{OH}}}^{-}$ and ${{{\text{H}}}_{3}{\text{O}}}^{+}$. The heat capacity of the ${\left({\text{MSA}}\right)}_{{\text{m}}}\cdot {\left({{\text{H}}}_{2}{\text{O}}\right)}_{{\text{n}}}$ clusters increases with the addition of ${\text{MSA}}$ or ${{\text{H}}}_{2}{\text{O}}$ molecules, indicating enhanced molecular vibration and hydrogen bonding network. The calculated chemical shifts align well with the experimental data, with better agreement observed in the measured ^1^H-NMR spectra of ${\text{MSA}}\cdot {\left({{\text{H}}}_{2}{\text{O}}\right)}_{n} , \left(m=1-2, n=3-5\right)$, particularly at lower hydration levels.

## Data Availability

The data that support the findings of this study are available from the corresponding author on reasonable request.
